# A multi‐omics analysis for the prediction of neurocognitive disorders risk among the elderly in Macao

**DOI:** 10.1002/ctm2.909

**Published:** 2022-06-13

**Authors:** Yan Han, Xingping Quan, Yaochen Chuang, Qiaoxing Liang, Yang Li, Zhen Yuan, Ying Bian, Lai Wei, Ji Wang, Yonghua Zhao

**Affiliations:** ^1^ State Key Laboratory of Quality Research in Chinese Medicine Institute of Chinese Medical Sciences University of Macau Taipa Macao SAR China; ^2^ Kiang Wu Nursing College of Macau Macao Macao SAR China; ^3^ State Key Laboratory of Ophthalmology, Zhongshan Ophthalmic Center Sun Yat‐sen University Guangzhou China; ^4^ Department of Gastrointestinal Surgery Second Clinical Medical College of Jinan University, Shenzhen People's Hospital Shenzhen China; ^5^ Centre for Cognitive and Brain Sciences University of Macau Taipa Macao SAR China; ^6^ School of Traditional Chinese Medicine Beijing University of Chinese Medicine Beijing China

**Keywords:** exosomes, gut microbiota, multi‐omics, neurocognitive disorders

## Abstract

**Background:**

Due to the increasing ageing population, neurocognitive disorders (NCDs) have been a global public health issue, and its prevention and early diagnosis are crucial. Our previous study demonstrated that there is a significant correlation between specific populations and NCDs, but the biological characteristics of the vulnerable group predispose to NCDs are unclear. The purpose of this study is to investigate the predictors for the vulnerable group by a multi‐omics analysis.

**Methods:**

Multi‐omics approaches, including metagenomics, metabolomic and proteomic, were used to detect gut microbiota, faecal metabolites and urine exosome of 8 normal controls and 13 vulnerable elders after a rigorous screening of 400 elders in Macao. The multi‐omics data were analysed using R and Bioconductor. The two‐sided Wilcoxon's rank‐sum test, Kruskal–Wallis rank sum test and the linear discriminant analysis effective size were applied to investigate characterized features. Moreover, a 2‐year follow‐up was conducted to evaluate cognitive function change of the elderly.

**Results:**

Compared with the control elders, the metagenomics of gut microbiota showed that *Ruminococcus gnavus*, *Lachnospira eligens*, *Escherichia coli* and *Desulfovibrio piger* were increased significantly in the vulnerable group. Carboxylates, like alpha‐ketoglutaric acid and d‐saccharic acid, and levels of vitamins had obvious differences in the faecal metabolites. There was a distinct decrease in the expression of eukaryotic translation initiation factor 2 subunit 1 (eIF2α) and amine oxidase A (MAO‐A) according to the proteomic results of the urine exosomes. Moreover, the compound annual growth rate of neurocognitive scores was notably decreased in vulnerable elders.

**Conclusions:**

The multi‐omics characteristics of disturbed glyoxylate and dicarboxylate metabolism (bacteria), vitamin digestion and absorption and tricarboxylic acid cycle in vulnerable elders can serve as predictors of NCDs risk among the elderly of Macao. Intervention with them may be effective therapeutic approaches for NCDs, and the underlying mechanisms merit further exploration.

## Graphical abstract

- Multi‐omics characteristics of Macao elderly susceptible to neurocognitive disorders (NCDs) were investigated.
- Gut microbiota, faecal metabolites and urine exosomes were detected utilizing metagenomics, metabolomic and proteomic.
- Disturbed glyoxylate and dicarboxylate metabolism (bacteria), vitamin digestion and absorption and tricarboxylic acid cycle can serve as predictors of NCDs risk.

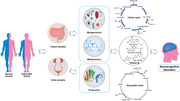

## 1 BACKGROUND

Cognitive function is one of the most basic activities of consciousness in humans and consists of several domains such as memory, attention and concentration, executive function and language/verbal skills.^1^ Neurocognitive disorders (NCDs) are characterized by a cognitive decline in one or more domains, including delirium and mild and major NCD following the latest Diagnostic and Statistical Manual of Mental Disorders‐V of the American Psychiatric Association.^2^, ^3^ NCDs affect nearly 50 million individuals worldwide, with more than 10 million new cases increasing each year.^4^ Alzheimer's disease (AD) is the most common NCDs, whose impairment will become increasingly evident as the population ages, with its prevalence likewise rising with age, from less than 1% of people under 60 to over 40% of people over 85.^5^ Due to the rapid global population ageing, NCDs have become a major public health challenge.

The intestinal microbiota has been proved to play a key role in influencing central nervous functions, including emotional responses and behaviour, and its metabolites may also cause neurodegenerative diseases through the gut–brain axis.^6^ Alterations in the composition of gut microbiota are associated with the development of NCDs. Studies showed that the distribution of intestinal microbiota gradually shifts from non‐pathogenic to pathogenic facultative anaerobic bacteria with increasing age.^7^ In addition, many studies have also shown that urine compositions can serve as potential biomarkers in the early diagnosis of NCDs. Disturbances in phospholipid and amino acid metabolism, alterations in the l‐glutamine and 5‐l‐glutamylglycine, palmitic amide and lysophosphatidylcholine metabolites all occurred in the urine of patients with early AD.^8^ Urine Alzheimer‐associated neuronal thread protein (AD7c‐NTP) can accurately predict amyloid‐beta protein deposition in the brain of AD patients, and increased AD7c‐NTP in cerebrospinal fluid and urine is correlated with AD severity positively.^9^, ^10^ Urine exosomes are protein‐containing vesicle, which can facilitate the transfer of proteins, lipids and nucleic acid to mediate intercellular communication. The role of proteins in exosomes determined to be involved in pathological process of many diseases, including AD.^11^ Gut microbiota had influence on host urine proteins expression and host‐secreted proteins influenced gut microbiota composition likewise.^12^

In our previous study on the investigation of susceptibility to NCDs in Macao elderly individuals, we recruited 400 older adults from elderly healthcare centres randomly and found that there was a vulnerable population which belonged to Yin‐deficient constitution of Chinese medicine contributed to the decline of neurocognitive function, especially visual space dimension.^13^ To discover the biochemical basis of the vulnerable population predisposed to NCDs for an early prediction of NCDs occurrence, multi‐omics approaches, including metagenomics, metabolomic and proteomic were employed to investigate gut microbiota, faecal metabolites and urine exosomes from the elderly. In addition, follow‐ups after 2 years of the vulnerable elders were conducted to assess their alteration of cognitive function level.

## 2 METHODS

### 2.1 Study design and subjects

**FIGURE 1:**
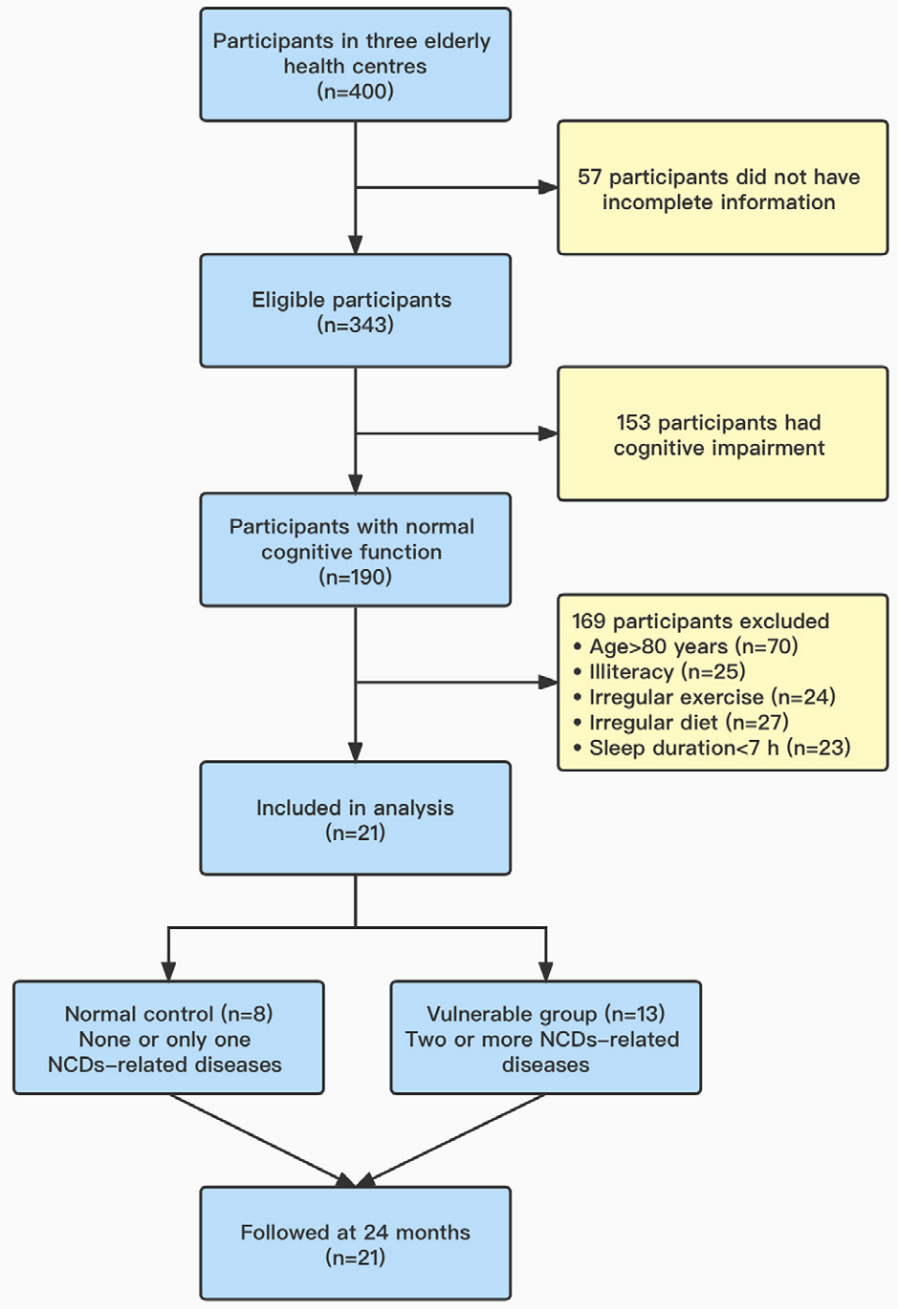
Flow chart of study inclusion/exclusion criteria

Recruitment for the trial was conducted in Macao from September to December 2019, and the follow‐up period of the trial was 24 months. Three elderly health centres were randomly selected from three administrative regions of Macao (Peninsula, Taipa and Coloane), and 400 participants were randomly selected from elders aged 65 years and above in these elderly health centres, and 57 participants were excluded because of incomplete information. A total of 21 elderly individuals were finally included in the study, who met the inclusion criteria (Figure 1). The inclusion criteria for the study were (1) Chinese residents who have lived in Macao for more than 10 years, (2) no intellectual and language communication barriers, able to understand and answer the questions in Cantonese, and (3) no suffering from major diseases of heart and/or lung in the past year. Exclusion criteria were (1) elders with cognitive impairment, (2) age >80 years old, (3) illiteracy, (4) irregular exercise, (5) irregular diet, (6) sleep duration <7 h and (7) antibiotics administration within 2 weeks. Participants who suffered from tumour, heart failure, mental illness and other serious systemic diseases and could not complete the questionnaire even with assistance were excluded. The elders were grouped in accordance with the number of chronic diseases susceptible to the occurrence of NCDs: “normal control (*n* = 8)” with none or only one chronic disease, whereas “vulnerable group (*n* = 13)” for those with two or more chronic diseases susceptible to the occurrence of NCDs, such as diabetes, hypertension and hyperlipidaemia. The Hong Kong version of Montreal Cognitive Assessment was used to evaluate neurocognitive scores of subjects from various domains.^14^ Power and sample size calculations were performed in Power and Sample Size (HyLown Consulting LLC; http://powerandsamplesize.com/). A two‐group time‐to‐event analysis involved comparing the time it took for NCDs to occur between two groups (Cox PH, 2‐Sided Equality).^15^ The elders were followed up after 2 years to reassess their neurocognitive scores and compare the compound annual growth rate (CAGR) from 2019 to 2021 between the two groups.

### 2.2 Samples collection

Fresh faecal and first morning urine from the elderly within two groups were collected at the healthcare centres. A protease inhibitor cocktail (P1005, Beyotime, Nanjing, China) and antibiotics (Pen‐Strep, Gibco, Invitrogen, San Diego, CA, USA) were added to avoid proteolysis and bacterial growth in the urine samples. The faecal and urine samples were processed within 4 h after collection in the laboratory and stored at minus 80°C until analysis. Total DNA was extracted from frozen stools using the QIAamp PowerFecal Pro DNA Kit (QIAGEN, 51804). The DNA quantity and purity were assessed using Thermo Scientific's NanoDrop One. Urine exosomes isolation was performed through differential ultracentrifugation as previously described.^16^ In brief, 30‐ml urine samples were centrifuged at 500×*g* (15 min) and 17 000×*g* (45 min) to remove the cellular debris and large membrane vesicles. The supernatants were then pelleted at 200 000×*g* for 65 min at 4°C (70 Ti Rotor, Beckman Coulter). The exosome pellets were resuspended in 30 ml of phosphate‐buffered saline.

### 2.3 Metagenomic sequencing of intestinal microbiota

The DNA metagenomic shotgun sequencing of the stool samples was performed as previously described.^17^ In brief, the VAHTS Universal DNA library Prep Kit for Illumina (Vazyme, Nanjing, China) was used to prepare sequencing libraries and the KAPA SYBR FAST qPCR Kit (Kapa Biosystems, Wilmington, MA, USA) was used to assess quantity by qPCR. Paired‐end 2×150‐bp sequencing was performed on a NovaSeq 6000 instrument (Illumina, San Diego, CA, USA). After FastQC quality control, the sequence reads were pre‐processed with the removal of human reads by HiSAT2 and DeconSeq to obtain clean non‐human sequences. The relative abundance was represented by the ratio of the total mapped reads of each species, normalized by the total mapped microbial reads and the genome size within each sample. The HMP Unified Metabolic Analysis Network (HUMAnN2) was used to analyse the abundance of microbial BioCyc pathways. The diversity and the difference of bacterial communities between the control and vulnerable groups were assessed using α‐ and β‐diversity, respectively. α‐Diversity was assessed by Chao1, Simpson and Shannon index, and β‐diversity was assessed by principal coordinate analysis (PCoA). To identify the significant different species, the linear discriminant analysis (LDA) effective size (LEfSe) was conducted.

### 2.4 Non‐targeted metabolomics of faecal sample

Faecal metabolites were extracted with methanol and analysed by the ultra‐high‐performance liquid chromatography–tandem mass spectrometry (UHPLC–MS/MS) for non‐targeted metabolomics analysis. The Compound Discoverer 3.1 was used to perform peak alignment, peak picking and quantitation for each metabolite. The accurate qualitative and relative quantitative results were obtained according to matched peaks with the mzCloud, mzVault and Mass List database. Data were collected in both positive and negative electrospray modes. The scan rate was one scan per second with a capillary voltage of 3500 V. The molecular features of the samples were obtained using the Mass Hunter Qualitative Analysis Software (Agilent Technologies).^18^ The Kyoto Encyclopedia of Genes and Genomes (KEGG) database, Human Metabolome Database (HMDB) and Lipid Metabolites and Pathways Strategy (LIPID MAPS) database were employed to annotate metabolites. The metabolites with variable importance in the projection (VIP) > 1 and *p* < .05 and fold change ≥2 or FC ≤ .5 were considered to be differential metabolites according to principal components analysis (PCA) and partial least squares discriminant analysis (PLS‐DA) performed at metaX.^19^ Volcano plots were used to filter metabolites of interest based on log2 (fold change) and −log10 (*p* value) of metabolites by ggplot2 in R language.

### 2.5 Proteomic analysis of urine exosomes

Proteomic analyses were performed as previously described using the label‐free proteomics method.^20^ In brief, 100‐μg protein for each sample was digested and then the peptide was desalted by a Phenomenex Strata‐X C18 SPE column. Then, the sample was fractionated by high pH reverse‐phase HPLC with an Agilent 300Extend‐C18 column. Peptides were subjected to an NSI source followed by tandem mass spectrometry (MS/MS) in Q Exactive coupled online to the UPLC. The electrospray voltage applied was 2300 V. Intact peptides were detected in the orbitrap at a resolution of 60 000. Peptides were selected for MS/MS using an normalized collision energy (NCE) setting of 28, and ion fragments were detected in the orbitrap at a resolution of 15 000. For MS scans, the *m*/*z* scan range was 400–1200. The fixed first mass was set as 100 *m*/*z*. Differentially expressed proteins were identified with a cut‐off of absolute fold change ≥1.5. Tandem mass spectra were searched against the Swiss‐Prot Human database. Gene Ontology (GO) analysis was performed to classify all identified proteins into cell components, molecular function and biological process using the UniPort‐GOA database, InterProScan and GO annotation.

### 2.6 Statistical analysis

The GraphPad Prism 8 and R software 3.6 were used for statistical analyses. Two‐sided Wilcoxon's rank‐sum test was used for comparisons of α‐ and β‐diversity of gut microbiota between the control and vulnerable groups. The box edges denoted the first and third quartiles and the horizontal line denoted the median for all boxplots, with the whiskers extending up to 1.5‐fold interquartile ranges. The LEfSe method and the Kruskal–Wallis rank sum test were performed to identify features characterizing significant differences. A value of LDA > 2 and *p* < .05 were considered statistically significant. Analysis of differential metabolites expression was performed using *p* values and VIP values. Metabolites with values of *p* < .05 and VIP > 1 were regarded as potential biomarkers. The receiver‐operating characteristic curve analysis was used to evaluate the efficiency of different metabolites predictors of vulnerable group. Correlation analysis among metagenomic, metabolomic and proteomics data was undertaken using protein and metabolites identified as significantly different. Spearman multi‐omics correlations were calculated using R and the Benjamini–Hochberg method was used to control the false discovery rate. For metabolomics and proteomics data analysis, multiple hypothesis correction with the Benjamini–Hochberg method was applied as well. The ggplot2 package was used to perform visual presentation of multi‐omics correlations. A value of *p* < .05 was considered significant difference.

## 3 RESULTS

### 3.1 Sociodemographic features and neurocognitive scores of subjects

The study samples consisted of 21 elders 65 years of age or above screened by living habits, clinical history, no major diseases of heart and/or lung, no intellectual and communication barriers. Matched samples included 8 subjects (females) in the normal control group and 13 subjects in vulnerable group (3 males and 10 females). The ratio of gender, age, education and neurocognitive scores of the two groups are shown in Table 1. No differences were found among the parameters within the two groups.

**TABLE 1:** Sociodemographic features and neurocognitive scores of the study subjects

Variable	Normal control	Vulnerable group	*p*‐Value^*^
**Gender**			
Men	0	3	.505
Women	8	10	
**Age**	71.25 ± 3.11	73.46 ± 3.80	.183
**Education**			
Primary schools and below	8	11	.243
Middle school and above	0	2	
**Neurocognitive scores**	22.13 ± 3.91	24.69 ± 3.23	.118

*Notes*: Data are expressed as mean ± SD except where frequencies are used for categorical data.

^*^ Fisher's exact test chi‐squared test for categorical variables; *t*‐test for continuous variables.

### 3.2 Differential gut microbiota compositions and pathways prediction

**FIGURE 2:**
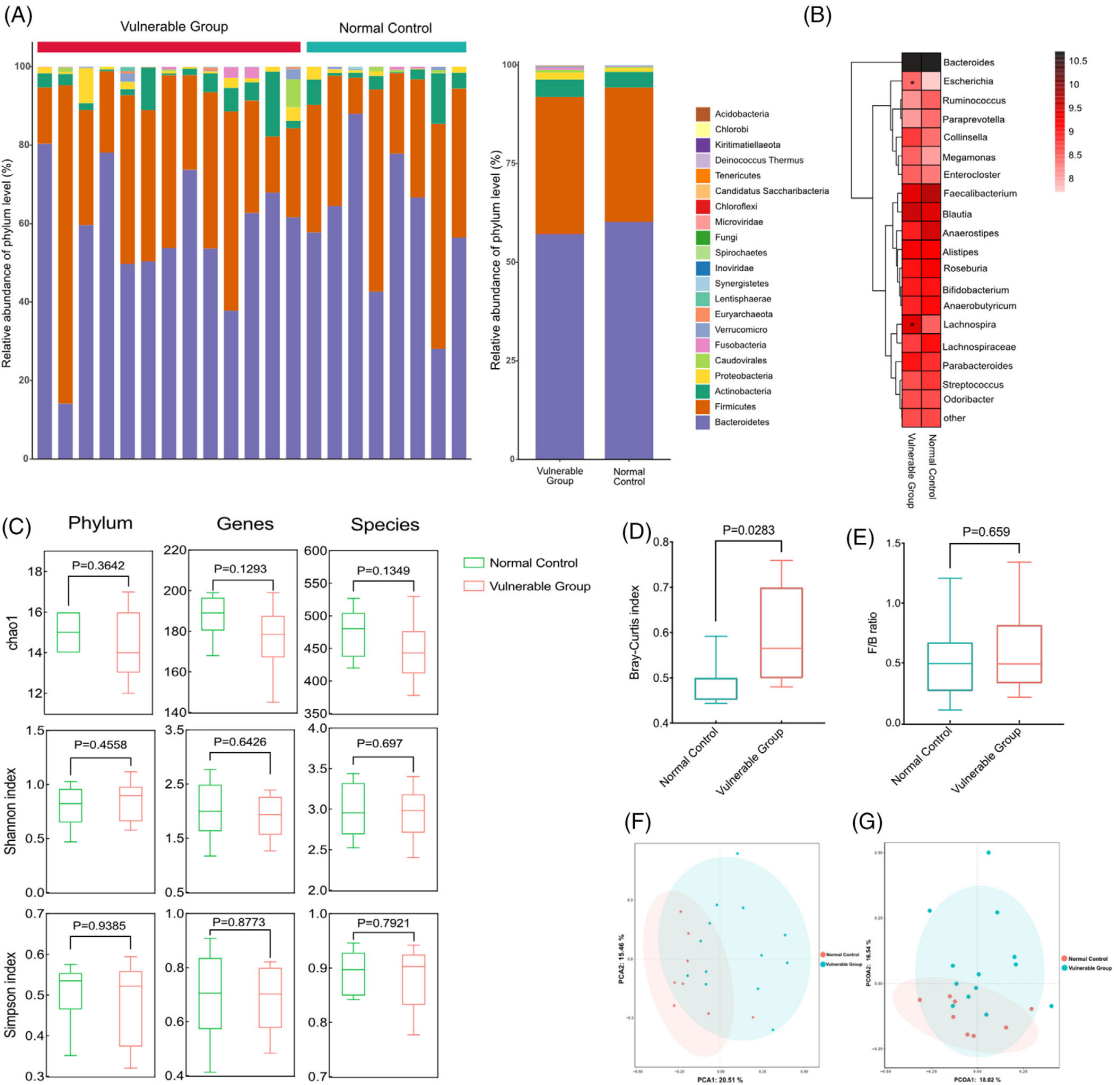
Gut microbiota analysis in the vulnerable group: (A) the relative abundance of gut microbiota at the phylum level between the control and vulnerable groups. (B) Heatmap analyses of gut microbiota at the genus level in top 20. (C) Chao1 index, Shannon index and Simpson index in α‐diversity analysis at the phylum, genus and species levels. (D) β‐Diversity measured with Bray–Curtis dissimilarity. *p* Values of α‐ and β‐diversity were computed using a two‐sided Wilcoxon test. (E) *Firmicutes*/*Bacteroidetes* (F/B) ratio between the control and vulnerable groups. (F) PCA of the gut microbiota at the species level. (G) PCoA of gut microbiota based on species‐level Bray–Curtis distance. PCA, principal component analysis; PCoA, principal coordinate analysis

At the phylum level, the gut microbiota composition of the two groups was dominated by *Bacteroidetes*, *Firmicutes*, *Actinobacteria* and *Proteobacteria*, which on average accounted for up to 98% of the relative abundance. Compared with the control group, *Firmicutes*, *Actinobacteria* and *Proteobacteria* were increased whereas *Bacteroidetes* was decreased at the phylum level in the vulnerable group (Figure 2A). The top 20 genera heatmap with the highest abundance for in‐depth analysis showed that *Escherichia* was increased and *Lachnospira* was decreased significantly in the vulnerable group compared with the control group (Figure 2B). α‐Diversity analysis was estimated based on the Chao1, Shannon and Simpson index, which reflected the richness and diversity of microbiota. As shown in Figure 2C, the diversity indices at the phylum, genus and species levels were decreased in the vulnerable group, but this difference did not achieve statistical significance. β‐Diversity analysis was used to evaluate the variance of diversity between two groups and was assessed with Bray–Curtis dissimilarities. The Bray–Curtis dissimilarities within the vulnerable group were significantly higher than the control groups (Figure 2D). Although the *Firmicutes*/*Bacteroidetes* (F/B) ratio was increased in the vulnerable group compared with the control group, the change was not statistically significant (Figure 2E). The PCA (Figure 2F) and the PCoA (Figure 2G) of species composition showed a clear delineation between the control and vulnerable groups, suggesting that the dysbiosis of gut microbiome was associated with vulnerable group.

**FIGURE 3:**
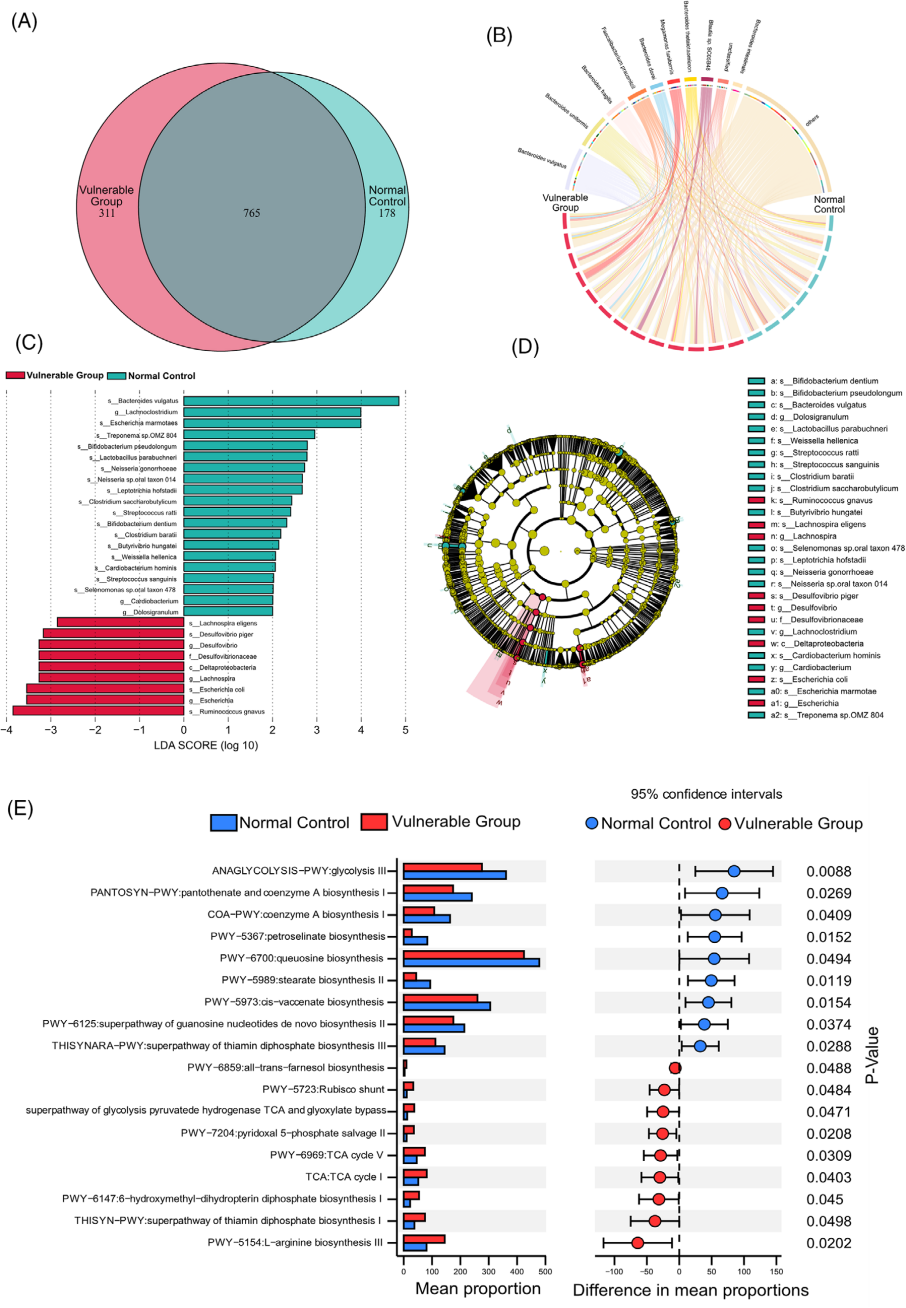
Significant shifts in gut microbial compositions at species levels and bacterial functional profiles: (A) Venn diagram of gut microbiota. (B) The Chord diagram of gut microbiota at the species level. (C) Distinctive gut microbiota composition associated with the vulnerable group revealed by LDA effect size (LEfSe) analyses, with LDA score >2. (D) Cladogram of the LEfSe analysis of gut microbiota. (E) The comparative analysis for relative abundances of BioCyc pathways between the control and vulnerable groups. STAMP analysis was applied to identify significant differential abundant BioCyc pathways. LDA, linear discriminant analysis

In addition, the Venn diagram showed unique and common species of gut microbiota to better understand their shared richness. This analysis showed that 765 operational taxonomic units (OTUs) accounting for the total richness were common to all the samples, whereas 178 OTUs and 311 OTUs accounted for control and vulnerable groups, respectively (Figure 3A). The Chord diagram showed the ten most enriched gut microbiota species and the linkage between species and the two groups. The arcs indicated connections, represented proportionally by the size of each arc. Node segments along a circle represented species and the node size indicated the abundance of contributing species (Figure 3B). The differential microbiota of the control and vulnerable groups were presented based on LEfSe analysis (Figure 3C). There were 20 bacterial taxa enriched in the normal control, which consisted of *Bacteroides vulgatus*, *Escherichia marmotae*, *Treponema* sp. OMZ 804, *Bifidobacterium pseudolongum*, *Lactobacillus parabuchneri*, *Neisseria gonorrhoeae*, *Neisseria* sp. oral taxon 014, *Leptotrichia hofstadii*, *Clostridium saccharobutylicum*, *Streptococcus ratti*, *Bifidobacterium dentium*, *Clostridium baratii*, *Butyrivibrio hungatei*, *Weissella hellenica*, *Cardiobacterium hominis*, *Streptococcus sanguinis*, *Selenomonas* sp. oral taxon 478 at the species level, and *Lachnospira*, *Cardiobacterium*, *Dolosigranulum* at the genus level. Moreover, a total of nine bacterial taxa were enriched in the vulnerable group, which included *Lachnospira eligens*, *Desulfovibrio piger*, *Escherichia coli*, *Ruminococcus gnavus* at the species level, and *Desulfovibrio* (genus), *Escherichia* (genus), Desulfovibrionaceae (family), *Desulfovibrionales* (order) and *Deltaproteobacteria* (class). Cladogram was obtained from the LEfSe analysis, indicating the phylogenetic distribution of microbiota (Figure 3D).

PICRUSt was used to predict the metagenome functional content based on metagenomic shotgun sequencing and BioCyc pathways analysis. BioCyc pathways were a variety of biological processes regulated as a unit, which were constructed based on information provided in the BioCyc database. STAMP analysis revealed 18 pathways with significant differences between the 2 groups, including 5 pathways of generation of precursor metabolites and energy, 4 pathways of vitamins biosynthesis, 3 pathways of fatty acid biosynthesis, 2 pathways of Coenzyme A (CoA) biosynthesis, 1 pathway each of nucleic acid processing, nucleoside and nucleotide biosynthesis, secondary metabolite biosynthesis and amino acid biosynthesis. Compared with the control group, the abundance of glycolysis and CoA biosynthesis was significantly reduced in the vulnerable group. Moreover, the superpathway of thiamine diphosphate biosynthesis was enriched in the control group. Thiamine diphosphate, also known as vitamin B_1_, plays an important role in the energy metabolism.

### 3.3 Differential faecal metabolites

**FIGURE 4:**
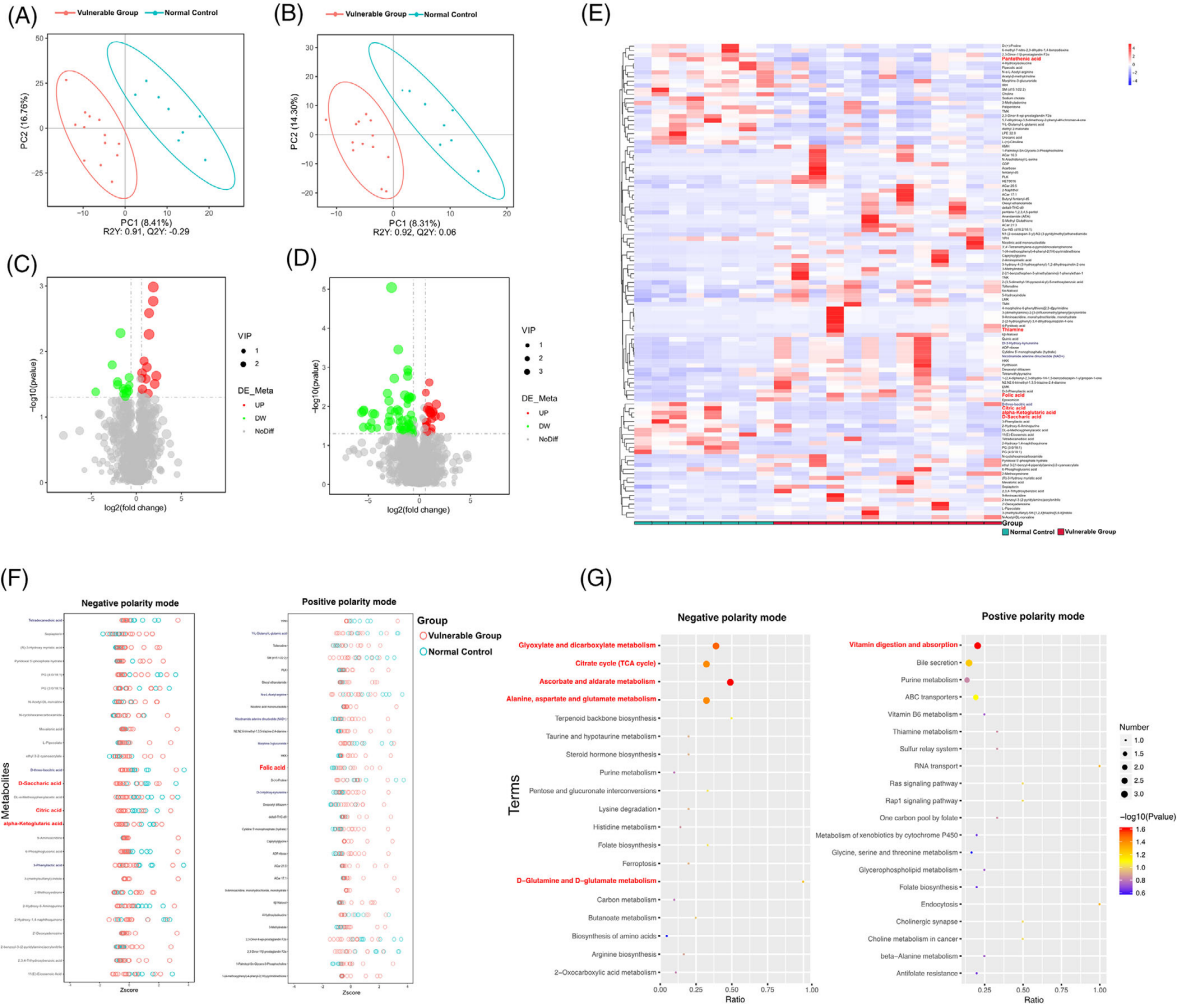
Untargeted analysis of the faecal metabolome: (A) PLS‐DA score plot of primary metabolites in negative ion mode. (B) PLS‐DA score plot in positive ion mode. (C) Volcano map of differential metabolites in negative–positive ion mode between the control and vulnerable groups, green: downregulated metabolites; red: upregulated metabolites. (D) Volcano map of differential metabolites in positive ion mode. (E) Heat maps of significant differential metabolites. (F) *z*‐Score plot of the top 30 differentially expressed metabolites in negative and positive ion mode, each circle represents a sample. (G) Comparing the bubble plot of the top 20 significantly enriched KEGG pathways between the control and vulnerable groups in negative and positive ion mode. PLS‐DA, partial least squares discriminant analysis

To evaluate the metabolic changes in gut microbiome, the faecal samples were analysed by UHPLC–MS/MS. The PLS‐DA analysis was used to explore the metabolic differences between the two groups, and the cross‐validation of PLS‐DA models of all multi‐omics data had done to avoid data overfitting (Figure S4). The results suggested that the metabolite distribution in the vulnerable group was different from that in the control group both in negative ion (Figure 4A) and positive ion models (Figure 4B). Compared with the control group, 14 and 13 metabolites were significantly upregulated and downregulated in the negative ion model, respectively (Figure 4C), and 58 and 24 metabolites were significantly upregulated and downregulated, respectively, in the positive ion model (Figure 4D). All upregulated and downregulated differential metabolites had been screened, and the hierarchical clustering analysis clearly classified the metabolites with the same and different characteristics between the control and vulnerable groups. The results were visualized in a heatmap, as shown in Figure 4E. To delineate changes in metabolites further, the top 30 metabolites were listed based on *p*‐value, and *z*‐score plots were constructed. As shown in Figure 4F, carboxylates were more downregulated in the vulnerable group, including alpha‐ketoglutaric acid, d‐saccharic acid, eicosenoic acid, 6‐phosphogluconic acid, tetradecanedioic acid and anandamide. The utilization of carboxylates was an important source of nutrients and energy. The vitamins levels were increased in faecal samples of the vulnerable group, including folic acid, thiamine and 4‐pyridoxic acid, whereas pantothenic acid was decreased.

**FIGURE 5:**
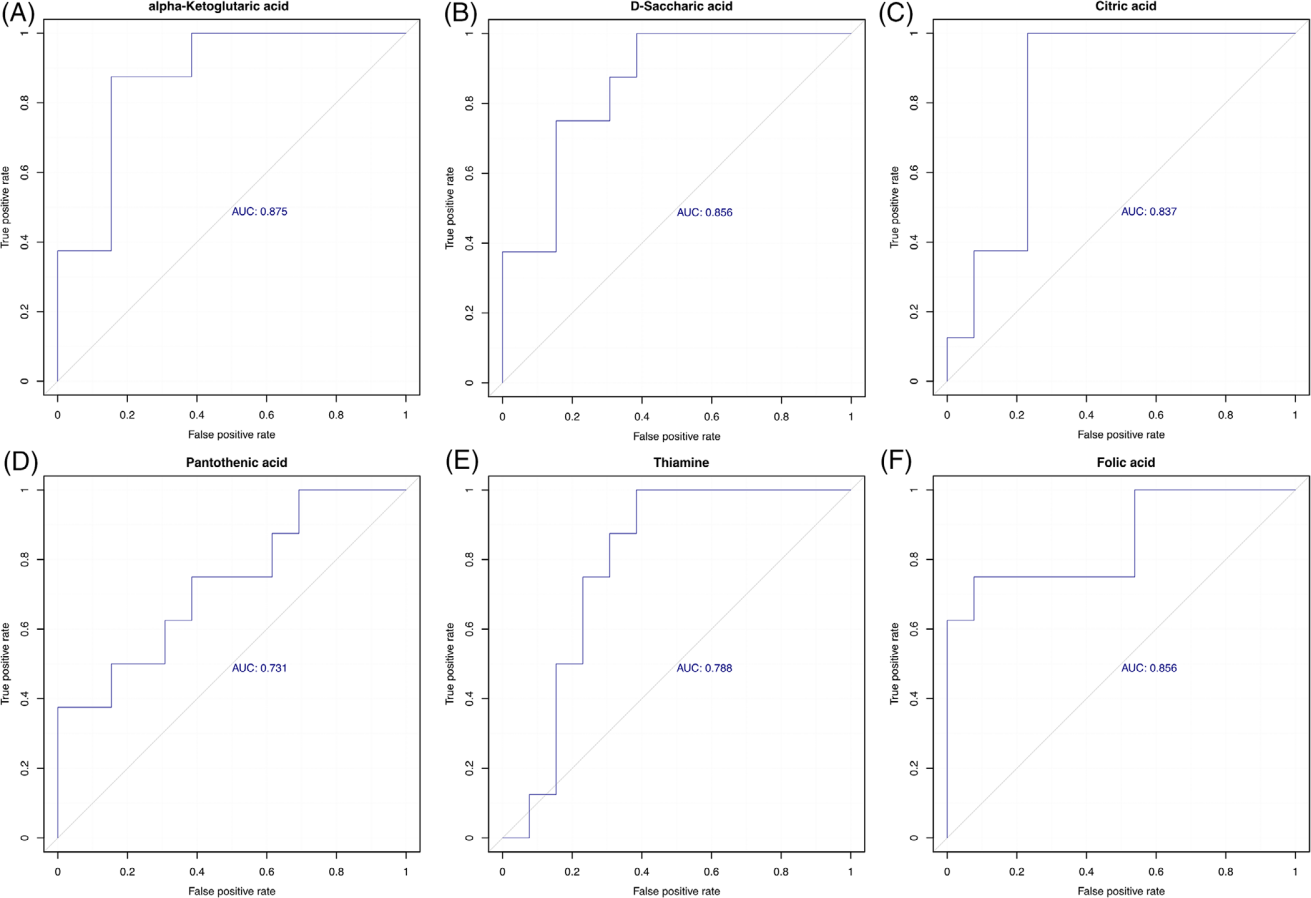
ROC curve of differential metabolites predicting vulnerable group susceptible to NCDs: (A) ROC curve of alpha‐ketoglutaric acid, (B) ROC curve of d‐saccharic acid, (C) ROC curve of citric acid, (D) ROC curve of pantothenic acid, (E) ROC curve of thiamine, (F) ROC curve of folic acid. NCD, neurocognitive disorder; ROC, receiver operating characteristic

KEGG annotation analysis was used to find all pathways of differential metabolites. Further metabolic pathway analysis, including enrichment analysis and topological analysis, 39 key pathways that were the most relevant to metabolite difference had been screened, the first 6 lines of which are shown in Table 2, and which were shown as a bubble plot in Figure 4G. The area under curve (AUC) values for alpha‐ketoglutaric acid, d‐saccharic acid and citric acid predicting vulnerable group were .875, .856 and .873, respectively. Likewise, the AUC values for pantothenic acid, thiamine and folic acid were .731, .788 and .856, respectively (Figure 5A–F). Alpha‐ketoglutaric acid was found to be the most significant predictor for vulnerable elders. Table 2 shows that alpha‐ketoglutaric acid was the common differential metabolite in the top six metabolic pathways enriched in differential metabolites, and it might be the most critical differential metabolite. The results showed that ascorbate and aldarate metabolism, glyoxylate and dicarboxylate metabolism, vitamin digestion and absorption, tricarboxylic acid (TCA) cycle, alanine, aspartate and glutamate metabolism and d‐glutamine and d‐glutamate metabolism pathway were high correlation with differential metabolites.

**TABLE 2:** Metabolic pathways analysis (top 6)

Metabolic pathways	*p‐*Value	Enriched differential metabolites
Ascorbate and aldarate metabolism	.011	Alpha‐ ketoglutaric acid, d‐saccharic acid
Glyoxylate and dicarboxylate metabolism	.018	Alpha‐ketoglutaric acid, citric acid
Vitamin digestion and absorption	.025	Pantothenic acid, thiamine, folic acid
TCA cycle	.027	Alpha‐ketoglutaric acid, citric acid
Alanine, aspartate and glutamate metabolism	.027	Alpha‐ketoglutaric acid, citric acid
d‐Glutamine and d‐glutamate metabolism	.047	Alpha‐ketoglutaric acid

### 3.4 Differential urine exosomes proteins

**FIGURE 6:**
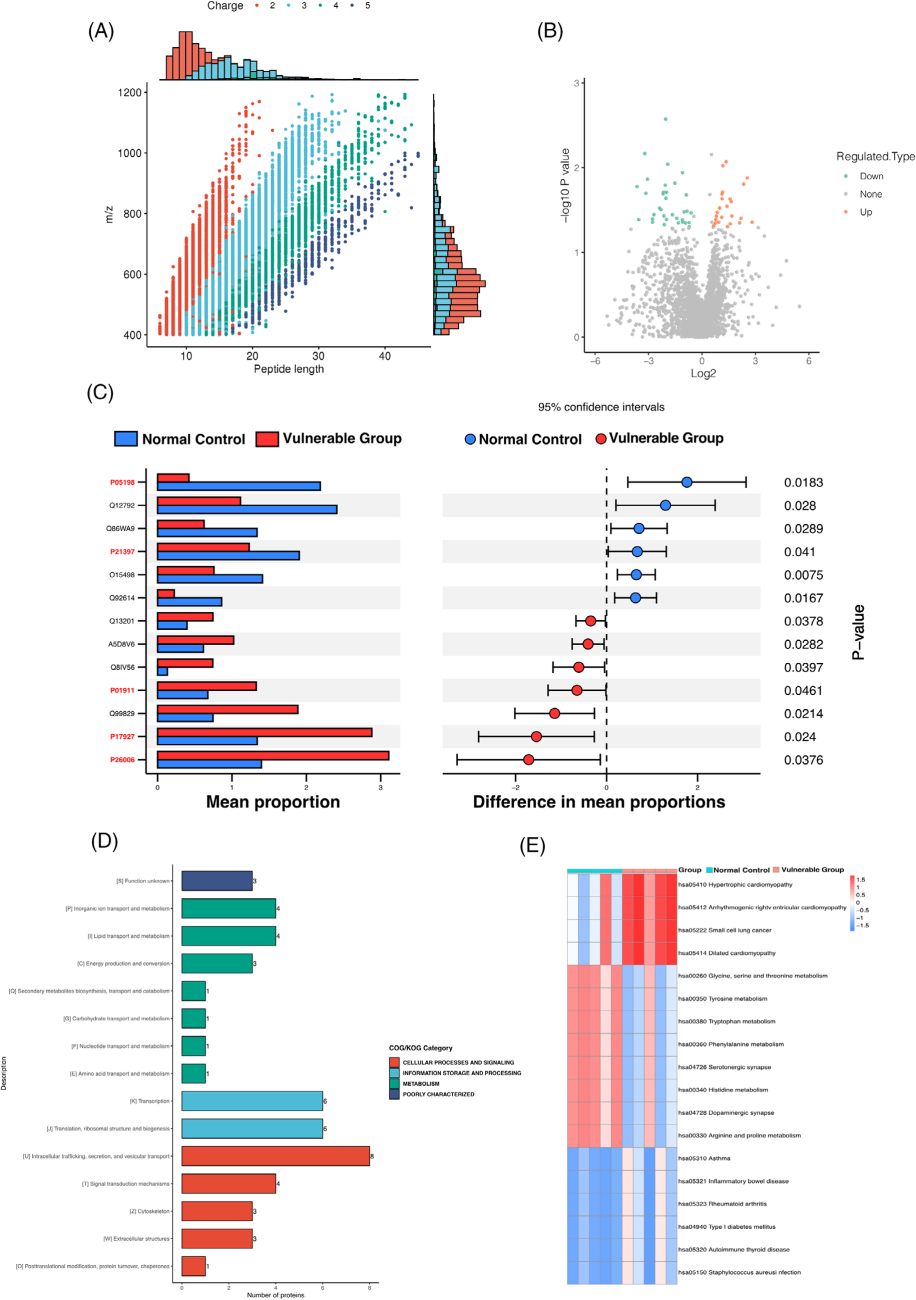
Label‐free quantitative proteomics analysis of urine exosomes: (A) length distribution of all identified peptides. (B) Volcano map of differential proteins between the control and vulnerable groups, green: downregulated metabolites; red: upregulated metabolites. (C) The comparative analysis for the relative abundances of proteins between the control and vulnerable groups by STAMP. (D) KOG function classification of significant differential proteins. (E) Heatmap of differential abundant KEGG pathways identified in the control and vulnerable groups. KOG, euKaryotic Orthologous Groups

To define if there were differences in urine exosomes proteins content in the control and vulnerable groups, the purified exosomes from samples were subsequently analysed. A total of 2448 944 secondary spectrograms were obtained by mass spectrometry, and 388 572 spectrograms were available for analysis. A total of 23 147 peptide segments were identified, among which 22 165 were specific segments. Most of the peptides were distributed in 7–20 amino acids and met the quality control requirements (Figure 6A). In total, 3306 proteins were identified, of which 2712 were quantifiable. The screening criteria for differential abundance of proteins were fold‐change >1.5 (upregulated) or <1.5 (downregulated) and *p* < .05. As shown in the volcano map, red was used to indicate prominent upregulated differential proteins, and blue represented apparent downregulated differential proteins. Compared with the control group, 24 and 34 proteins were significantly upregulated and downregulated, respectively (Figure 6B). STAMP analysis revealed 13 proteins with significant differences between the 2 groups. Compared with the control group, the eukaryotic translation initiation factor 2 subunit 1 (eIF2α, P05198) was the most significantly decreased protein in the vulnerable group. Amine oxidase flavin‐containing A (MAO‐A, P21397) was downregulated in the vulnerable group as well, which had important functions in the metabolism of vasoactive and neuroactive amines in the central nervous system and peripheral tissues. In addition, HLA class II histocompatibility antigen (P01911), integrin alpha‐3 (P26006) and complement receptor type 1 (P17927) were significantly upregulated in the vulnerable group (Figure 6C).

For all annotated proteins involved eukaryotic homologous protein clusters assignment, the EuKaryotic Orthologous Groups (KOGs) classification was shown in Figure 6D. KOG annotation sorted all the proteins into four major categories. For the cellular processes and signalling category, the largest number of proteins was classified into the “Intracellular trafficking, secretion, and vesicular transport” term (eight proteins). Twelve proteins belonged to the information storage and processing category. For the metabolism category, the main terms were “inorganic ion transport and metabolism” (four proteins), “lipid transport and metabolism” (four proteins) and “energy production and conversion” (three proteins). Function annotation of KEGG pathways was performed to understand the function and bioprocess of the differentially expressed proteins based on the KEGG database. In the heatmap of differentially abundant KEGG pathways, enriched disease‐related pathways were upregulated in the vulnerable group, involving hypertrophic cardiomyopathy (hsa05410), arrhythmogenic right ventricular cardiomyopathy (hsa05412), small cell lung cancer (hsa05222) and dilated cardiomyopathy (hsa05414). The most upregulated pathways in the control group were the following: glycine, serine and threonine metabolism (hsa00260), tyrosine metabolism (hsa00350), tryptophan metabolism (hsa00380), phenylalanine metabolism (hsa00360), serotonergic synapse (hsa04726), histidine metabolism (hsa00340), dopaminergic synapse (hsa04728) and arginine and proline metabolism (hsa00330) (Figure 6E).

### 3.5 Correlations among differential gut microbiota, faecal metabolites and urine exosomes proteins

**FIGURE 7:**
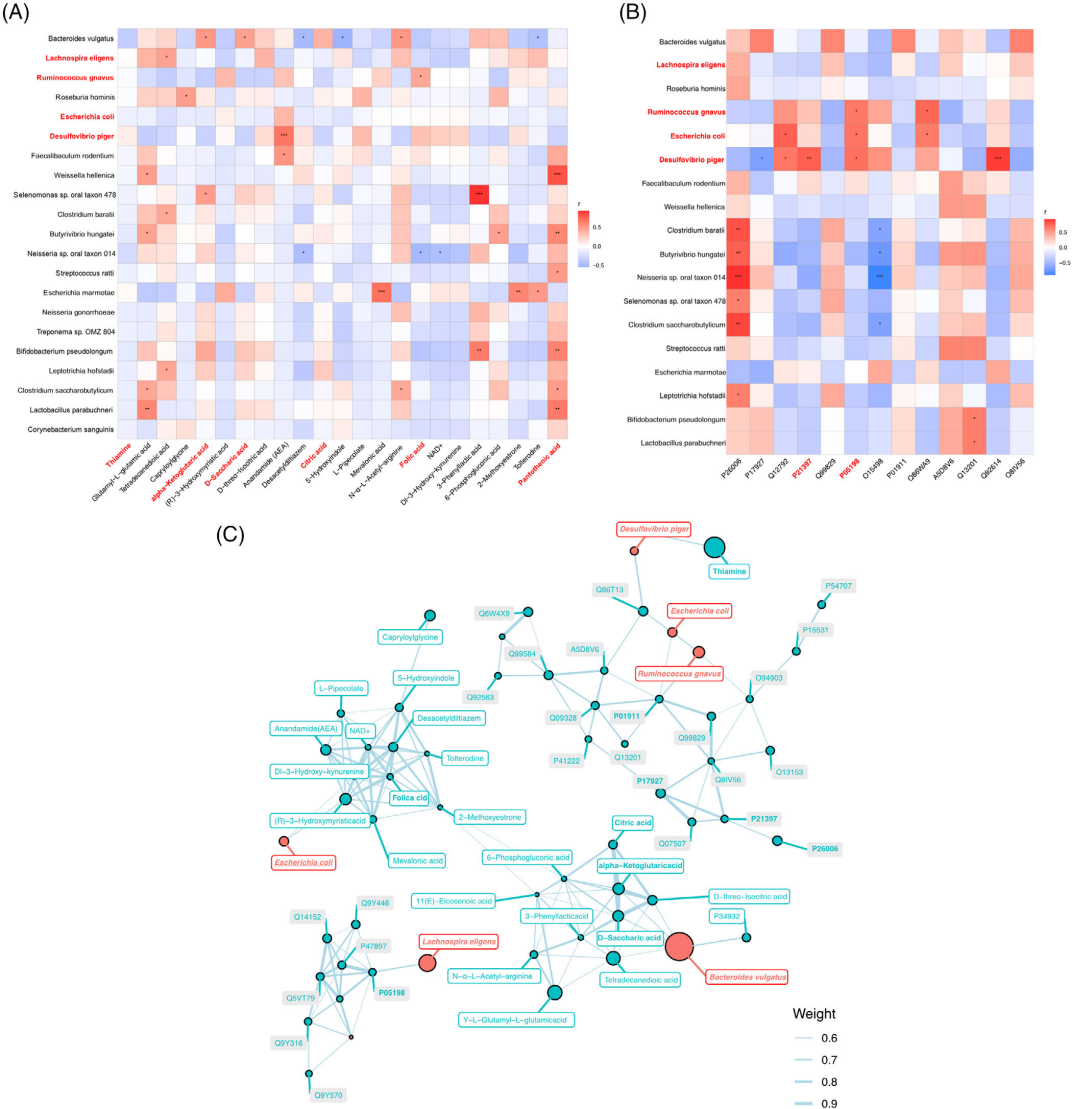
Associations of gut microbial changes with metabolome and proteome: (A) Spearman's rank correlation between differential gut microbiota species and faecal metabolites. (B) Spearman's rank correlation between differential gut microbiota species and urine exosomes proteins. (C) Co‐occurrence network of the microbiome, metabolome and proteome. The *r*‐value is represented by the gradient colour; red indicates positive correlation, and blue colour indicates negative correlation. ^*^ *p *< .05, ^**^ *p *< .01

The Spearman correlation analysis was used to investigate the relevance between the differential bacterial species and the faecal metabolites (Figure 7A). The amount of *B. vulgatus* was significantly correlated with several metabolites, such as alpha‐ketoglutaric acid, d‐saccharic acid and *N*‐α‐l‐acetyl‐arginine. The correlation of *R. gnavus* and folic acid, *L. eligens* and *Υ*‐l‐glutamyl–l‐glutamic acid, *D. piger* and anandamide (AEA) were significant as well (|*r*| > .5, *p* < .05, .01). We further correlated the bacterial species and urine exosomes proteins using the Spearman correlation analysis, and the result showed that the eIF2α (P05198) was significantly positively with *R. gnavus*, *E. coli* and *D. piger*; in addition, MAO‐A, P21397 showed significant positive correlations with *D. piger* as well (|*r*| > .5, *p* < .05, .01) (Figure 7B). To explore potential reciprocal interactions among altered gut microbiota, faecal metabolites and urine exosomes proteins, a co‐occurrence network was constructed based on the Spearman correlation analysis. We found that *R. gnavus*, *L. eligens*, *E. coli* and *D. piger* formed strong co‐occurring relationships with faecal metabolites and urine exosomes proteins involved within glyoxylate and dicarboxylate metabolism, vitamin digestion and absorption and TCA cycle. These correlations suggested that changes in gut microbiota composition and related metabolites were tightly related to hosting proteins and metabolism.

### 3.6 Two‐year follow‐up assessments for NCDs occurrence

To identify high susceptibility to NCDs in the vulnerable group, follow‐up was performed 2 years after the initial assessment of neurocognitive scores. In the control group, the cognitive function of all the elderly was normal, whereas 3 of 13 elders had transferred into mild NCD in the vulnerable group. Moreover, we calculated the CAGR of neurocognitive scores to assess the relative growth of vulnerable‐related neurocognitive scores. The CAGR formula is equal to [(*Y*/*X*)^(1/3)^ − 1] × 100%. Compared with the control group, the CAGR in the vulnerable group was significantly decreased, even presented negative growth as shown in Table 3, suggesting that their cognitive function would continue to deteriorate over time. Additionally, there existed the differences of gut microbiota species and faecal metabolites between the three subjects before they progressing to mild NCD and others in the vulnerable group (Figure S1).

**TABLE 3:** The CAGR of the control group and vulnerable group

Group	Initial scores (*X*)	Neurocognitive function	Follow‐up scores (*Y*)	Neurocognitive function	CAGR	The average CAGR
Normal control	16	Normal	20	Normal	7.72	.35 ± 5.50
	28	Normal	23	Normal	−6.35	
	22	Normal	21	Normal	−1.53	
	19	Normal	22	Normal	5.01	
	19	Normal	23	Normal	6.58	
	24	Normal	20	Normal	−5.90	
	24	Normal	24	Normal	0	
	25	Normal	23	Normal	−2.74	
Vulnerable group	27	Normal	22	Normal	−6.60	−3.77 ± 3.03^*^
	26	Normal	24	Normal	−2.63	
	28	Normal	20	Mild NCD	−10.61	
	23	Normal	23	Normal	0	
	22	Normal	18	Normal	−6.47	
	17	Normal	15	Mild NCD	−4.08	
	23	Normal	22	Normal	−1.47	
	26	Normal	22	Mild NCD	−5.42	
	28	Normal	24	Normal	−5.01	
	28	Normal	27	Normal	−1.20	
	27	Normal	25	Normal	−2.53	
	23	Normal	21	Normal	−2.99	
	23	Normal	23	Normal	0	

*Notes*: Data are expressed as mean ± SD.

Abbreviations: CAGR, compound annual growth rate; NCD, neurocognitive disorder.

^*^ *p *< .05 (unpaired *t*‐test).

## 4 DISCUSSION

Our previous study found the association of vulnerable population (Yin‐deficient constitution) with NCDs in Macao elderly individuals.^13^ To further investigate how the vulnerable elders interacted with NCDs, we considered the microbiome first. The term microbiome is suggested to describe the collective genome of human body microbiota, and it can be divided into three types depending on dominant genera, named enterotypes, including enterotype 1 (*Bacteroides*), enterotype 2 (*Prevotella*) and enterotype 3 (*Ruminococcus*).^21^ The classification of enterotypes has potential clinical implications, and it is useful in identifying the disease state of an individual to guide treatment and help in understanding different therapeutic measurements. Moreover, enterotype can be employed as an indicator of risk or susceptibility to a specific state of the body.^22^ To some extent, vulnerable population can be analogized to a specific enterotype.

The *Bacteroidetes* is a very diverse bacterial phylum, and its members mainly colonize in the colon. *Bacteroidetes* plays an important role in the development of the gastrointestinal tract and maintaining a healthy gut. In addition, *Bacteroidetes* can interact with the immune system for the activation of T‐cell‐mediated responses and produce butyrate, which is thought to have anti‐tumour properties.^23^, ^24^ Higher dietary fibre would favour the development of the *Bacteroidetes*, which can degrade complex plant polysaccharides and produce succinic acid, acetic acid and propionic acid.^25^ Previous studies have reported that the increased *Firmicutes*/*Bacteroidetes* (F/B) ratio would reduce polysaccharide metabolism and SCFA production.^26^ Our study found that *Bacteroidetes* was decreased, and the F/B ratio tended to increase in the vulnerable group, suggesting they might be linked to microbiota metabolic capacity reduction. At the genus level, the decrease of *Lachnospira* in vulnerable group was consistent with clinical research about gut microbiome features of Chinese patients newly diagnosed with AD or mild cognitive impairment.^27^ The link between altered gut microbiota composition and cognition has been investigated in various animal models, including germ‐free animals, antibiotics‐induced pseudo‐germ‐free animals and faecal microbiota transplants (FMT).^28^ The results of our study showed that the α‐diversity of gut microbiota leaned to decrease, and the β‐diversity was increased significantly in vulnerable elders compared with normal elders, the same as the changes in gut microbiota composition may cause cognitive decline.

Based on the LEfSe analysis, we identified four species, including *E. coli*, *L. eligens*, *D. piger* and *R. gnavus* as the potential gut microbiota markers for the vulnerable group. When it comes to the correlation between the four specific species and NCDs, the transplantations of *E. coli* isolated from the elderly can cause colitis and cognitive decline in mice, and *E. coli* might be associated with ageing‐dependent cognitive disorders.^29^ The increase of *Desulfovibrio* abundance coincided with abundant microglial accumulation at sites of amyloid deposition in the brain of AD mouse models, which was viewed as an inflammation‐related bacterial profile.^30^ *R. gnavus* was also found to be enriched in post‐operative cognitive dysfunction.^31^ These results suggested that the four specific species negatively influenced the cognitive function, and these enrichments accounted for the susceptibility of vulnerable group to NCDs partly.

The PICRUSt predicted metagenome function showed that glycolysis, CoA biosynthesis and the superpathway of thiamine diphosphate biosynthesis were significantly reduced in the vulnerable group compared with the control group. Glycolysis is the first studied pathway for the utilization of glucose, which is one of the major pathways of central metabolism. It is essential under all conditions of growth.^32^ CoA is a cofactor in a great number of enzymatic reactions and is crucial to intermediary metabolism, including oxidation of fatty acids, carbohydrates, and amino acids, and its derivatives are vital intermediates in energy metabolism. The biosynthesis of CoA is associated with the human neurodegenerative disorder with mutations in pantothenate kinase.^33^, ^34^ Thiamine diphosphate, a thiamine (vitamin B_1_) derivative, is also known to play a fundamental role in energy metabolism. It is an essential cofactor for a variety of enzymes such as pyruvate dehydrogenase, transketolase, pyruvate decarboxylase and α‐ketoglutarate dehydrogenase. A case–control study demonstrated that high thiamine diphosphate level is a protective factor for AD.^35^ The reduced function of these three pathways in the vulnerable group may result in NCDs someday.

The metabolomics results of the faecal sample showed that alpha‐ketoglutaric acid was the most significantly differential faecal metabolites in the vulnerable group. Alpha‐ketoglutaric acid, also referred to as alpha‐ketoglutarate (AKG), is one of two ketone derivatives of glutaric acid. It is a crucial intermediate in the TCA cycle.^36^ AKG can reverse dysfunctional mitochondria induced by oxidative stress. The oxidative stress impairs the ability of astrocytes to generate ATP, which is a common cerebral characteristic in AD patients.^37^ It has also been demonstrated that AKG also had significant neuroprotective effects by modulating the levels of reactive oxygen species in HT22 hippocampal neuronal cells.^38^ The AUC value in our study indicated that the predictive ability of alpha‐ketoglutaric acid for NCDs was reasonable in Macao elders.

The change in vitamins content is significant as well. A randomized controlled trial demonstrated that folic acid was beneficial to patients with AD by reducing inflammation.^39^ Moreover, a cross‐sectional study in the older hospitalized patients revealed that patients with dementia and delirium had lower whole blood thiamine compared to those without.^40^ 4‐Pyridoxic acid is the product of vitamin B_6_, and it was increased in serum of MCI (mild cognitive impairment) and AD patients.^41^ The main function of pantothenic acid, also called vitamin B_5_, is the synthesis of CoA and acyl carrier protein. A study had found that dietary intake of pantothenic acid was associated with cerebral amyloid burden in patients with cognitive impairment.^42^ Increase in folic acid, thiamine and 4‐pyridoxic acid, but decrease in pantothenic acid in the vulnerable group, may be due to vitamin digestion and absorption pathway difference. Besides the TCA cycle, both glyoxylate and dicarboxylate metabolism and ascorbate and aldarate metabolism belong to carbohydrate metabolism. Glyoxylate and dicarboxylate metabolism was associated with the TCA cycle via a connection with oxaloacetate. The lipid peroxidation caused by the accumulation of oxygen‐free radicals can impair synaptic interactions and cognitive function. Ascorbate and aldarate involved antioxidant defence mechanisms.^43^ Alanine, aspartate and glutamate metabolism, and d‐glutamine and d‐glutamate metabolism were both related to mitochondrial functions.^44^ Glutamate can be produced by several representing environmental bacteria or strains used in food fermentation and also be synthesized by lactic acid bacteria strains such as *Lactococcus lactis*, *Lactobacillus plantarum* and *Lactobacillus paracasei*.^45^, ^46^ Gut microbiota, including *Campylobacter jejuni* and *B. vulgatus*, affect glutamate metabolism. Moreover, l‐glutamate can be converted to d‐glutamate with glutamate racemase and gut bacteria, including *Brevibacterium avium*, *Brevibacterium lactofermentum* and *Corynebacterium glutamicum*.^47^

eIF2α and MAO‐A were both reduced in urinary exosomes protein in the vulnerable group according to the proteomics. It has been reported that eIF2α phosphorylation is significantly increased in the brains of different lines of APP/PS1 transgenic mice as well as AD patients.^48^ The environmental and genetic risks for AD may be associated with modulation of the eIF2α phosphorylation pathway because the accumulation of amyloid‐beta (Aβ) could induce eIF2α phosphorylation.^49^ It suggested that decreased eIF2α in the vulnerable group could be attributed to its phosphorylation. MAO‐A‐induced neurotransmitter alteration was related to AD directly and the activated MAO‐A involved the aggregation of Aβ and neurofibrillary tangles. It also impaired cognitive function through the damage of cholinergic neurons and the cholinergic system.^50^ Compared with the control group, a possible explanation for the decrease of MAO‐A in the urinary exosomes in vulnerable group was that with more activated MAO‐A in the brain, less would be metabolized in the urine.

The KEGG pathway analysis of enriched urinary exosomes proteins showed glycine, serine and threonine metabolism, tyrosine metabolism, tryptophan metabolism, phenylalanine metabolism, serotonergic synapse, histidine metabolism, dopaminergic synapse and arginine and proline metabolism were decreased in the vulnerable group. These results are following recent studies indicating that phenylalanine metabolism and arginine and proline metabolism presented downregulation in patients with AD and amnestic MCI.^51^ Besides, tyrosine metabolism can produce succinic acid involved the TCA cycle.^52^ AD patients showed a significant decreased activity of polyamine and tryptophan–kynurenine metabolisms.^53^ In addition, the AD mouse model suggested that the degeneration of ventral tegmental area dopaminergic neurons at pre‐plaque stages contributed to cognitive impairment and dysfunction of reward processing.^54^ Additionally, we expect further study can combine transcriptomics with proteomics approaches to test exosomes and discover more precise mechanisms of susceptible to NCDs elders.

Correlation analysis of multi‐omics is a good method to explore the predictors in the vulnerable group. In this study, we used the two‐by‐two correlation strategy to identify microbiome–metabolites and microbiome–proteins correlation pairs, respectively. Eventually, we obtained *R. gnavus* ‐folic acid‐eIF2α, *E. coli*‐eIF2α, *D. piger* ‐anandamide‐eIF2α and MAO‐A, *L. eligens* –Υ‐l‐glutamyl–l‐glutamic acid correlation pairs, which involved glyoxylate and dicarboxylate metabolism, vitamin digestion and absorption and TCA cycle. Compared with previous studies in other regions and countries, we find that there exists some similarity results in gut microbiota analysis for early prediction of NCDs. For example, the studies conducted in Singapore and the Netherlands also suggested that *Ruminococcus* was correlated with cognitive functions, which was regarded as risk indicators of MCI.^55^, ^56^ Moreover, most of the subjects in the studies were female, suggesting that *Ruminococcus* should exert a more predominant role for woman cognitive functions. Additionally, some research evidenced unique compositions in blood, for example, plasma Aβ42/40 and phosphorylated‐tau217 (Swedish longitudinal study), serum amyloid P, endothelin‐1 and interleukin‐2 (US study), C‐reactive protein (Finland study) and adiponectin (Spain study) contributed to a varying extent prediction of cognitive function evolution.^57^, ^58^, ^59^, ^60^ It is different from our findings from faecal metabolomic and urine exosomes proteomic analysis.

Interestingly, these multi‐omics characteristics in the vulnerable group are closely associated with the pathogenesis of NCDs, which is consistent with our previous cross‐sectional study. Hence, it is conceivably hypothesized that the vulnerable population is susceptibility to NCDs. Meanwhile, the 2‐year follow‐up outcomes supported the assumption, because the neurocognitive scores CAGR in vulnerable elders were significantly decreased. Although the small sample size is regarded as the limitations in the present study, the representability has still certain significance. Due to the complicated factors, for example, age, education level, habits of exercise and diet, and sleep duration, affecting the neurocognitive function in the elderly population, it caused only 21 samples were accordance with the research requirements after screening 400 elder individuals. In further studies, we will continue to expand the sample size, and conduct longitudinal studies to test the time course of alterations and their relationship with NCDs by the detection of relevant metabolites in plasma, specific phosphorylated proteins, and imaging technologies, such as functional MRI, which contributes to more comprehensive prediction for the vulnerable populations’ susceptibility to NCDs. Moreover, we compared the difference of gut microbiota abundance between enrolled elders already suffering from mild NCD and vulnerable subjects, and the results suggested that the abundances of four species of differential microbiota in mild NCD group had similar change trend to those in vulnerable group, which further validates the conviction of our differential microbiota findings (Figure S2). In addition, our initial experimental results also demonstrated that pseudo germ‐free aged C57 mice administered by the transplantation of faecal microbiota from NCDs donor suggested the decrease of escape latency in the 5‐day navigation test, indicating cognitive deficits (Figure S3). The concise mechanisms need to be defined by FMT in further study.

## CONFLICT OF INTEREST

The authors declare no financial or other conflicts of interest in this work.

## Supporting information

Supporting Information

## Data Availability

The data that support the findings of this study are available from the corresponding author upon reasonable request.
