# Supplementary material for: A multi‐omics analysis for the prediction of neurocognitive disorders risk among the elderly in Macao
Source: Clin Transl Med. 2022 Jun 13;12(6):e909. doi: 10.1002/ctm2.909 (PMC9191869; doi:10.1002/ctm2.909)
Supplement: Supplementary file 1 — Supporting Information [file CTM2-12-e909-s001.docx]

**Supporting information**

**A multi-omics analysis for** **prediction of neurocognitive disorders risk among the elderly in Macao**

Yan Han ^a^, Xingping Quan ^a^, Yaochen Chuang ^b^, Xingqiao Liang ^c^, Yang Li ^d^, Zhen Yuan ^e^, Ying Bian ^a^, Lai Wei ^c^, Ji Wang ^f^, Yonghua Zhao ^a, *^

^a^ State Key Laboratory of Quality Research in Chinese Medicine, Institute of Chinese Medical Sciences, University of Macau, Macao SAR 999078, China

^b^ Kiang Wu Nursing College of Macau, Macao, 999078, China

^c^ State Key Laboratory of Ophthalmology, Zhongshan Ophthalmic Center, Sun Yat-sen University, Guangzhou 510060, China

^d^ Department of Gastrointestinal Surgery, Second Clinical Medical College of Jinan University, Shenzhen People's Hospital, Shenzhen 518020, China

^e^ Centre for Cognitive and Brain Sciences, University of Macau, Macau SAR 999078, China

^f^ School of Traditional Chinese Medicine, Beijing University of Chinese Medicine, Beijing 100029, China

* Address correspondence to Yonghua Zhao, M.D., Research Building N22, Institute of Chinese Medical Sciences, University of Macau, Avenida da Universidade, Taipa, Macao SRA 999078, China.

Tel: +853 88224877 Fax: +853 28841358

Email: [yonghuazhao@um.edu.mo](mailto:yonghuazhao@um.edu.mo)

**Supporting materials and methods**

**Statistical power calculations of subjects**

Power calculations were performed in Power and Sample Size (HyLown Consulting LLC; http://powerandsamplesize.com/). We used this calculator to perform power and sample size calculations for a time-to-event analysis. A two-group time-to-event analysis involves comparing the time it takes for NCDs to occur between two groups (Cox PH, 2-Sided Equality).^1^ This calculator uses the following formulas to compute power:


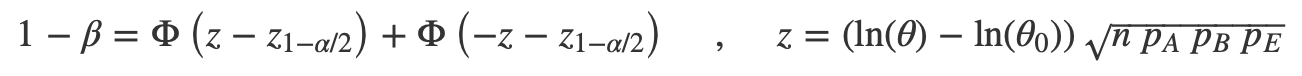


The calculator above and the formulas below use the notation that

- θ is the hazard ratio, which was 0.0498 in Macao.^2^
- ln(θ) is the natural logarithm of the hazard ratio.
- p_E_ is the overall probability of NCDs occurring within the study period.
- p_A_ and p_B_ are the proportions of the sample size allotted to the two groups, named 'A' and 'B'.
- n is the total sample size.
- Φ is the standard Normal distribution function.
- α is Type I error, 0.05.

β is Type II error, meaning 1−β is power.

**The processing of the** **illumina raw data**

The sequencing reads were first quality filtered using Trimmomatic v0.36 and PRINSEQ v0.20.4. Human reads were removed using KneadData v0.6.1 (https://bitbucket.org/biobakery/kneaddata). High quality non- human reads were mapped against a custom database using Kraken2 v2.0.9. A total of 29,943 complete microbial genomes were downloaded, of which 19,362 were bacterial, 368 were archaeal, 9,346 were viral, and 867 were fungal. The complete bacterial, archaeal, and viral genomes were downloaded from RefSeq database using the –download-library option of kraken2-build. The complete fungal genomes were manually downloaded from GenBank database. The results of taxonomic classification were filtered using a confidence score of 0.20. Only species with more than 10 reads in at least one sample were retained.^3^

**Faecal microbiota transplantation (FMT)**

Broad-spectrum antibiotics (ampicillin 1 g/L, neomycin sulfate 1 g/L, metronidazole 1 g/L) dissolved in drinking water were given to aged C57 mice for 14 consecutive days to construct pseudo germ-free mice. The drinking solution were renewed every 2 days. Fecal microbiota was prepared by diluting 1 g of fecal sample in 10 mL of sterile PBS.^4^ The fecal material was suspended, and 0.2 mL of the suspension from fecal samples of elderly people with NCDs and normal cognition was administered by gavage into each mouse recipient for 10 days, respectively. After 14 days FMT, the Morris water maze test was conducted to measure the cognitive function of mice.

**Supplemental references**

1. Chow S-C, Shao J, Wang H, Lokhnygina Y. *Sample size calculations in clinical research*. chapman and hall/CRC; 2017.

2. Iek Long L, Wen Z, Chin Ion L, Chong L, Hong Lei L. Macao Dementia Policy: Challenges and prospects (innovative practice). *Dementia (London)*. Feb 2021;20(2):791-795. doi:10.1177/1471301219887612

3. Liang Q, Li J, Zhang S, et al. Characterization of conjunctival microbiome dysbiosis associated with allergic conjunctivitis. *Allergy*. Feb 2021;76(2):596-600. doi:10.1111/all.14635

4. Zhan G, Yang N, Li S, et al. Abnormal gut microbiota composition contributes to cognitive dysfunction in SAMP8 mice. *Aging*. Jun 10 2018;10(6):1257-1267. doi:10.18632/aging.101464


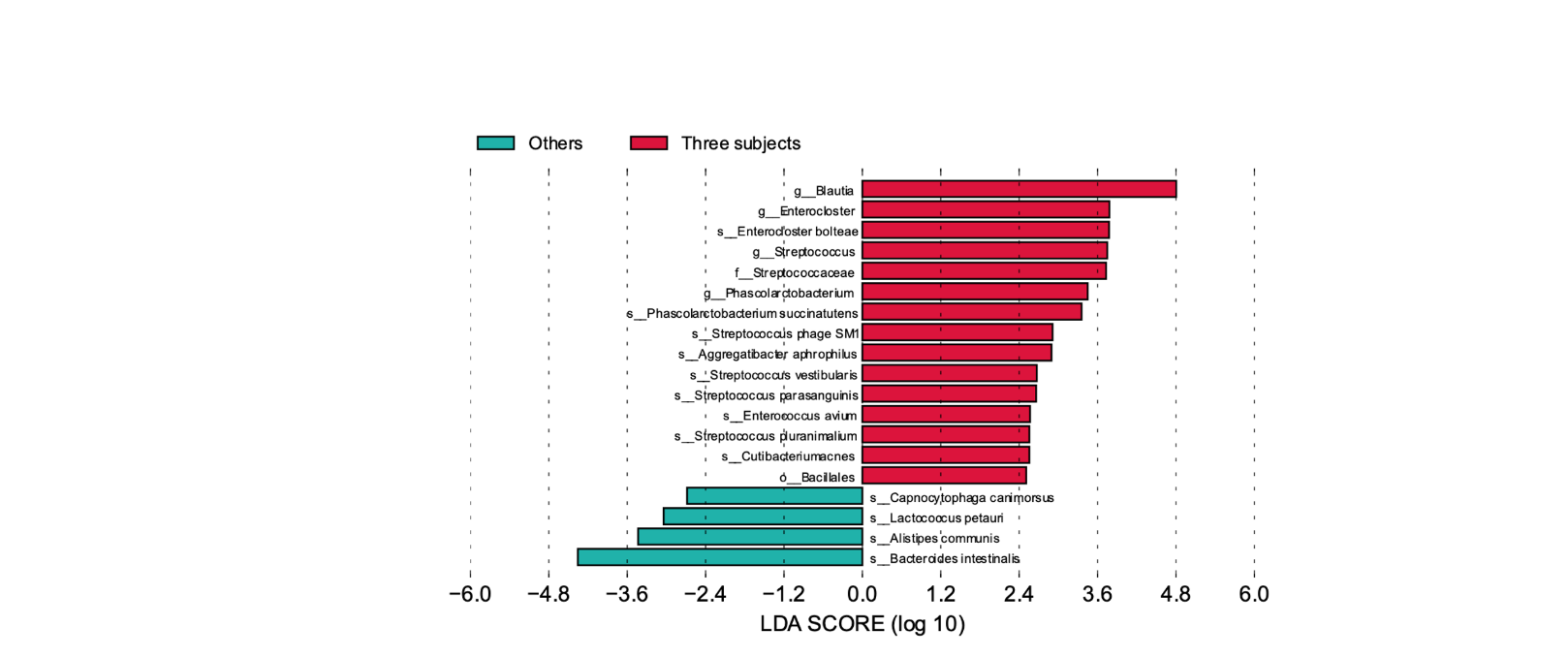


**Figure S1**. Distinctive gut microbiota composition of the three subjects before they processed to mild NCD is presented by linear discriminant analysis (LDA) effect size (LEfSe) analyses, with LDA score > 2.

**Figure S2.** The abundance comparison of four species of differential microbiota between in elders already suffering from mild NCD and vulnerble subjects. (a) *Lachnospira eligens,* (b) *Escherichia coli*, (c) *Desulfovibrio piger* and (d) *Ruminococcus gnavus*. Data are shown as mean ± SEM (normal control n = 8, vulnerable group n = 13, mild NCD n =11). ^*^*P* < .05, ^**^*P* < .01.

**Figure S3.** Escape latency (s) during the probe trial of the Morris Water Maze test. Data are shown as mean ± SEM (n = 10).

**Figure S4.** permutations plot of PLS-DA of (a) metagenomics data; (b) proteomics data; (c) metabolomic data in positive ion mode; (d) metabolomic data in negative ion mode.

**Figure S5.** The relative abundance of (a) *Escherichia* and (b) *Lachnospira* in the genus level. Data are shown as mean ± SEM (normal control n = 8, vulnerable group n = 13). ^*^*P* < .05.
